# The role of MRI in the diagnosis and management of tracheal diverticulum

**DOI:** 10.1186/s12880-022-00802-9

**Published:** 2022-04-22

**Authors:** Yuan Zhang, Yiqing Tan, Jing Chen, Cui Fang

**Affiliations:** Department of Radiology, Wuhan Third Hospital (Tongren Hospital of Wuhan University), Wuhan, 430000 China

**Keywords:** Tracheal diverticula, Paratracheal air cyst, MRI, CT

## Abstract

**Background:**

Multidetector CT is currently the best imaging method for detecting tracheal diverticulum (TD). Compared with CT, MRI is radiation-free and has higher resolution. However, the MRI characteristics of this disease have not been previously reported. The present retrospective study compared the MR and CT imaging features of TD, aiming to examine the role of MRI in TD diagnosis and management.

**Methods:**

Imaging data were collected in 26 TD patients divided into two groups, including the uninfected and infected groups. The MR and CT imaging features (size/wall/channel) of uninfected patients were compared. The performances of MRI and CT in diagnosing and monitoring therapeutic efficacy in infected TD patients were comparatively assessed.

**Results:**

The uninfected group comprised 25 cases with 25 lesions confirmed by CT, including 23 lesions (92%) detected by MRI, with an average diameter of 8.5 mm (range from 3 to 15 mm). Meanwhile, the average diameter was 7.8 mm as measured by CT (range from 2.8 mm to 14.7 mm). The lesion diameters of the two cases not detected by MRI were 2.3 mm and 2 mm. MRI detected walls of all the 23 lesions (23/23), while CT detected no wall (0/23). CT showed channels in 18 lesions (18/23) versus3 for MRI (3/23). The infected case presented with a paratracheal abscess; MRI clearly showed a relationship between the abscess and the trachea, while CT could not show the lesion source. MRI also sensitively showed the whole process of lesion absorption.

**Conclusions:**

MRI can be used as a supplementary method for TD diagnosis, providing information about the wall that cannot be obtained by CT. MRI is superior to CT in diagnosing infected TD cases presenting with a paratracheal abscess, and in monitoring therapeutic efficacy in these patients.

## Introduction

Paratracheal air cysts are incidental findings on CT examination of the chest and neck [1–4], which are almost always located at the right posterior aspect of the trachea [1, 2]. Several studies have suggested the majority of paratracheal air cysts are tracheal diverticulum (TD) [2, 5, 6], which is a benign condition characterized by one or multiple invaginations of the trachea wall [7]. Their incidence has steadily increased because of improvements in spatial resolution of multidetector CT and the application of thin-slices [8–10]. At present, multidetector CT is considered the best imaging method for detecting TD [11]. The MRI characteristics of this disease have not been previously reported. Indeed, the role of MRI in diagnosing and managing TD is unclear. However, MRI involves no radiation in contrast to CT, and may have some advantages over CT in managing TD patients and monitoring treatment efficacy in infected patients. Besides, several studies have shown that MRI has an important role in the diagnosis of juxtapupillary diverticulum [12, 13]. For this reason, the current study was designed. To our knowledge, no similar article has been previously reported.

## Materials and methods

This study was conducted with approval from the Institutional Review Board of our hospital. All procedures in the current study were performed in accordance with the ethical standards of the institutional and national research committees as well as with the Helsinki Declaration (as revised in 2013). Written informed consent was obtained from all patients.

From January 2020 to January 2022, all TD cases (236) detected by CT in our hospital were reviewed; among them, only 26 patients underwent MR examination and were enrolled in this study. They included 25 uninfected and 1 infected cases.

All uninfected cases were asymptomatic and detected incidentally. The mean patient age was 43.83 ± 8.48 years (range from 35 to 55 years). 17 patients were female and 8 were male.

The infected case was a 45-year-old woman, presenting with a fever, lower neck pain and swelling, and progressive dysphagia.

### Examination

All cases were confirmed by CT., and underwent MRI with a duration of less than 1 week after CT.

CT was performed on a 128-slice CT scanner (Discovery HD-670, General Electric Healthcare, Milwaukee, USA). Scan and reconstruction parameters were: detector collimation, 0.625 mm × 128; thickness, 0.625 mm; pitch, 1.0; reconstruction interval, 0.625 mm; matrix, 1024 × 1024; tube voltage, 120 kV; tube current, 400 mA (Table 1). After scanning, the raw data were transferred to the CT workstation (AW4.5 General Electric Healthcare) to generate axial thin-slice images with a thickness of 0.625 mm as well as MRP images. The infected case was administered nonionic contrast medium (370mgI/ml; Ultravist; Berlin, Germany) via the elbow vein with a double-tube power injector at a flow rate of 3 ml/s and a volume of 2 ml/kg body weight. Scanning was performed in 30 and 60 s before and after contrast injection, respectively.

**Table 1:** Protocol for CT

Parameter	Chest
Detector collimation	128 × 0.625
Section thickness (mm)	0.625
Section increment (mm)	0.625
Pitch	1
kV	120
mA	400
Image matrix	1024 × 1024
Scan range	Chest

MRI was performed on a 1.5 T unit (Magnetom Avanto; Siemens Medical Solutions, Erlangen, Germany) with a head and neck coil. Sequences and scan parameters were: axial T1WI (TR, 540 ms; TE, 20 ms; matrix, 320 × 224; FOV, 220 mm × 200 mm), axial T2WI (TR, 3340 ms; TE, 101 ms; matrix, 320 × 224; FOV, 220 mm × 200 mm), axial T2WI with turbo inversion recovery magnitude (T2-TIRM, TR, 4000 ms; TE, 30 ms; matrix, 320 × 224; FOV, 220 mm × 200 mm; TI, 180 ms), coronal T2-TIRM (TR, 4000 ms; TE, 30 ms; matrix, 320 × 224; FOV, 300 mm × 300 mm; TI, 180 ms), with 5-mm thickness and a 1-mm interval (Table 2).

**Table 2:** Protocol for MRI

Parameter	Axial T1WI	Axial T2WI	Axial T2-TIRM
TR (ms)	540	3340	4000
TE (ms)	20	101	30
TI (ms)	–	–	180
Matrix	320 × 224	320 × 224	320 × 224
FOV	220 × 200	220 × 200	220 × 200
Thickness (mm)	5	5	5
Interval (mm)	1	1	1

### Analytical methods

Two chest radiologists with 10 years of work experience reviewed the MR data to calculate the positivity rate. The examined features, including the diameter, wall, channel and density/signal of each TD lesion, were compared on axial images between the MR and CT groups.

Diameter measured in MRI = [diameter measured in (T1WI + T2WI + T2-Tirm)]/3.

### Treatment and review

Conservative treatment (intravenous antibiotic therapy, mucolytic agents, and physiotherapy) was selected for the infected TD patient. MRI was performed on days 2, 4, 7 and 21 of treatment to evaluate its role in the diagnosis and treatment of infected TD. CT was only performed after 21 days of treatment because of radiation.

## Results

**Fig. 1:**
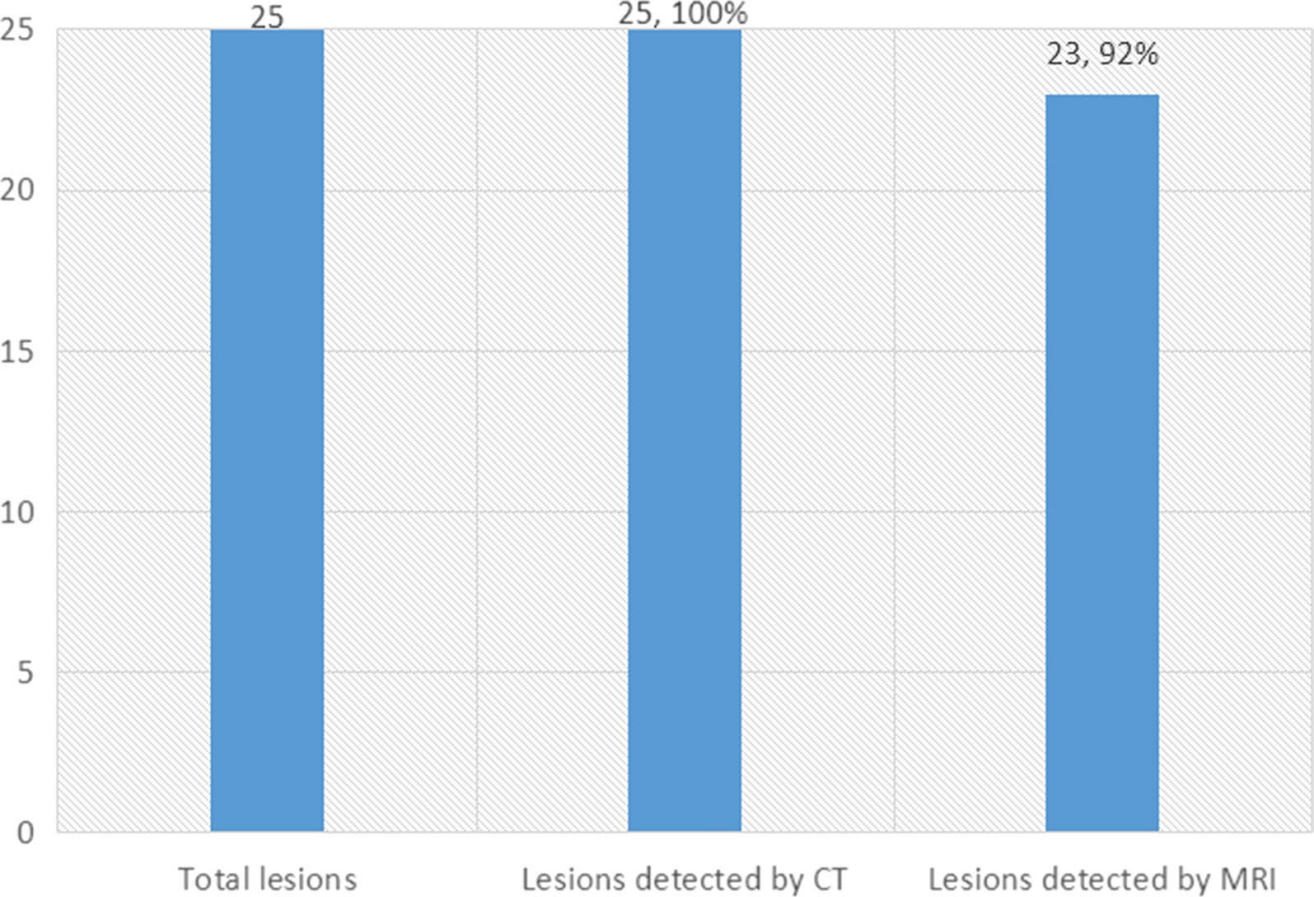
The uninfected group comprised 25 patients with 25 lesions confirmed by CT, including 23 lesions (92%) detected by MRI

**Fig. 2:**
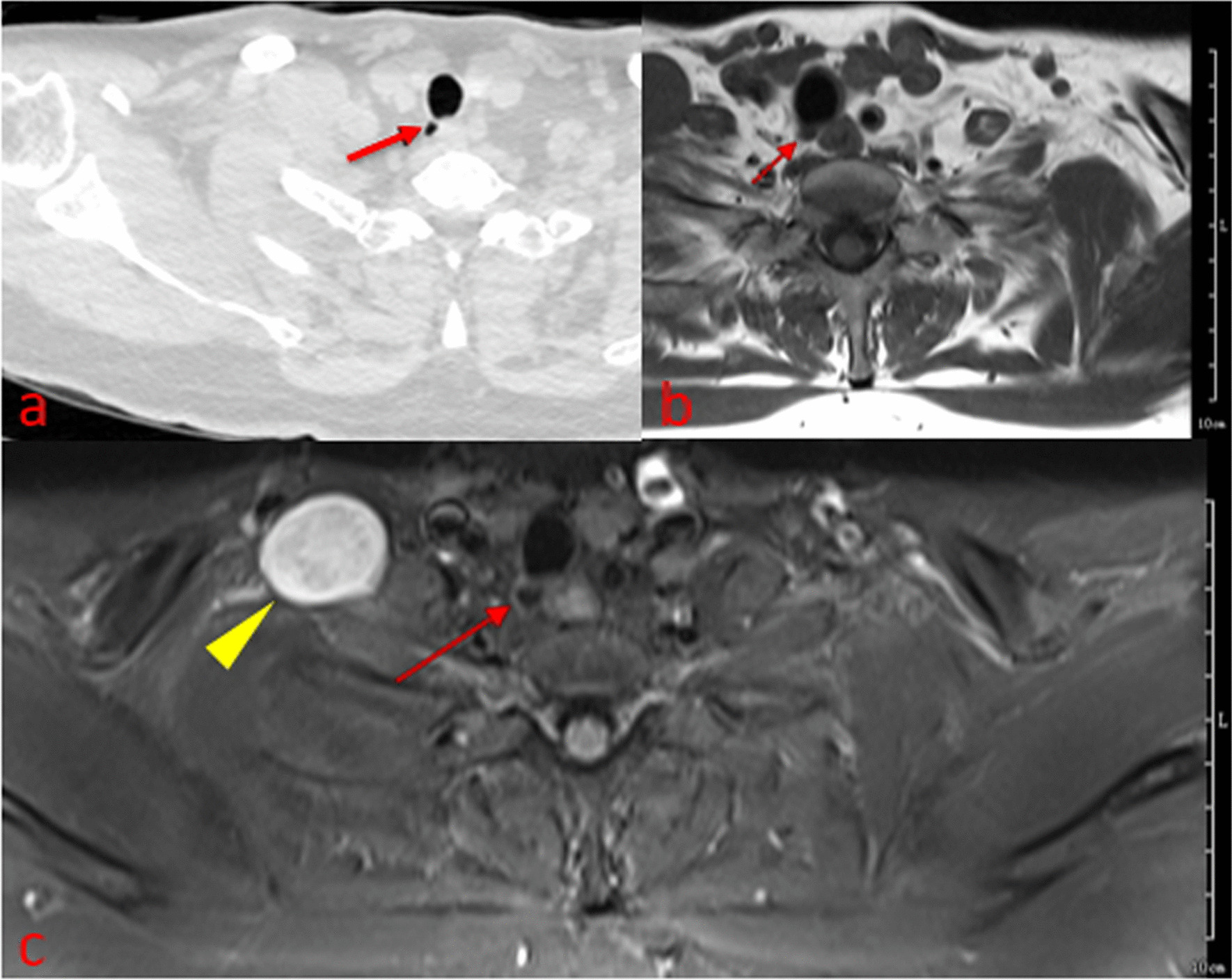
Axial CT image (**a**) in lung window showing a 5-mm diverticulum along the right posterolateral wall of the upper trachea (arrow). Axial T1WI (**b**) and T2-TIRM (**c**) showed a circular air-filled cystic lesion (no signal area) just behind the right side of the trachea (arrow). T2-Tirm clearly showed the slightly high signal wall of the lesion. Communication between the air cyst and the trachea was not seen in CT or MR images. A schwannoma in the right supraclavicular fossa is shown (arrowhead)

The uninfected group comprised 25 patients with 25 lesions, located on the right side, posterolateral to the trachea between the T1 and T3 vertebrae (confirmed by CT). Of these, 23 lesions were detected by MRI, indicating a positivity rate of 92% (Fig. 1). All lesions showed an oval or circular air-filled cystic region (air density cyst/no signal area) just behind the right side of the trachea (Fig. 2).

The average diameter of the 23 lesions was 8.5 mm (range from 3 to 15 mm) as measured by MRI *vs* 7.8 mm (range from 2.8 mm to 14.7 mm) as measured by CT (Table 3). Lesion diameters in the two cases not detected by MRI were 2.3 mm and 2 mm. MRI could detect lesion walls in all cases (iso-intensity in T1WI, and slight hyperintensity in T2WI and T2-TIRM), while CT could not detect them (Fig. 1). CT showed channels between the diverticulum and the trachea in 18 cases (18/23), versus only 3 (3/23) by MRI.

**Table 3:** Diameter of the TD

Measured by CT (mean, range)	7.8 mm (2.8–14.7 mm)
Measured by MRI (mean, range)	8.5 mm (3–15 mm)

**Fig. 3:**
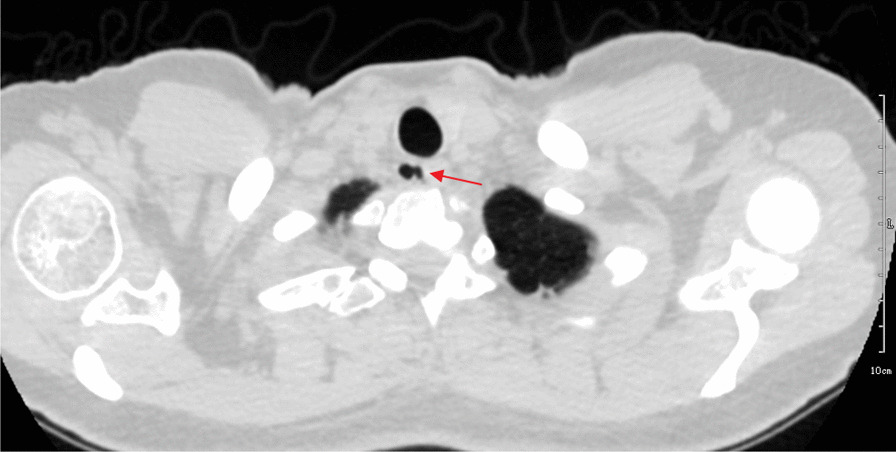
Axial CT image showing a tracheal diverticulum just behind the upper trachea (arrow)

**Fig. 4:**
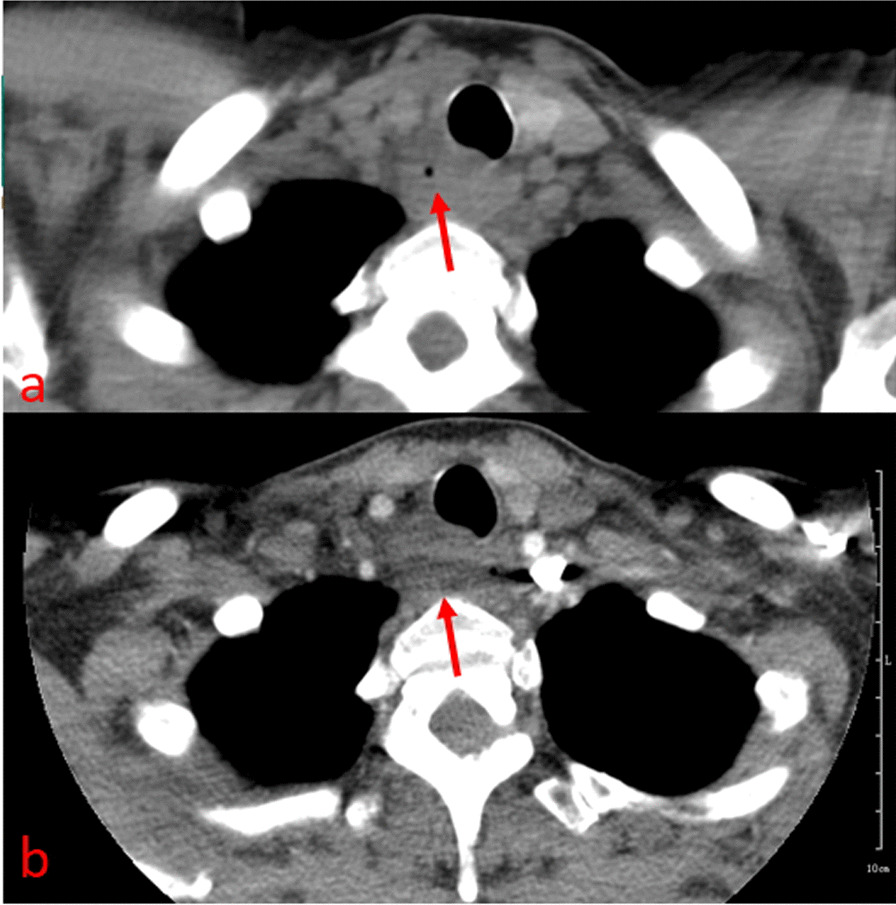
Axial CT image (**a**) showing a lower-density mass just behind the upper trachea (arrow). The small bubble inside the lesion suggests it may be an abscess. No enhancement was found after contrast injection (**b**). CT could not identify the origin of the lesion

**Fig. 5:**
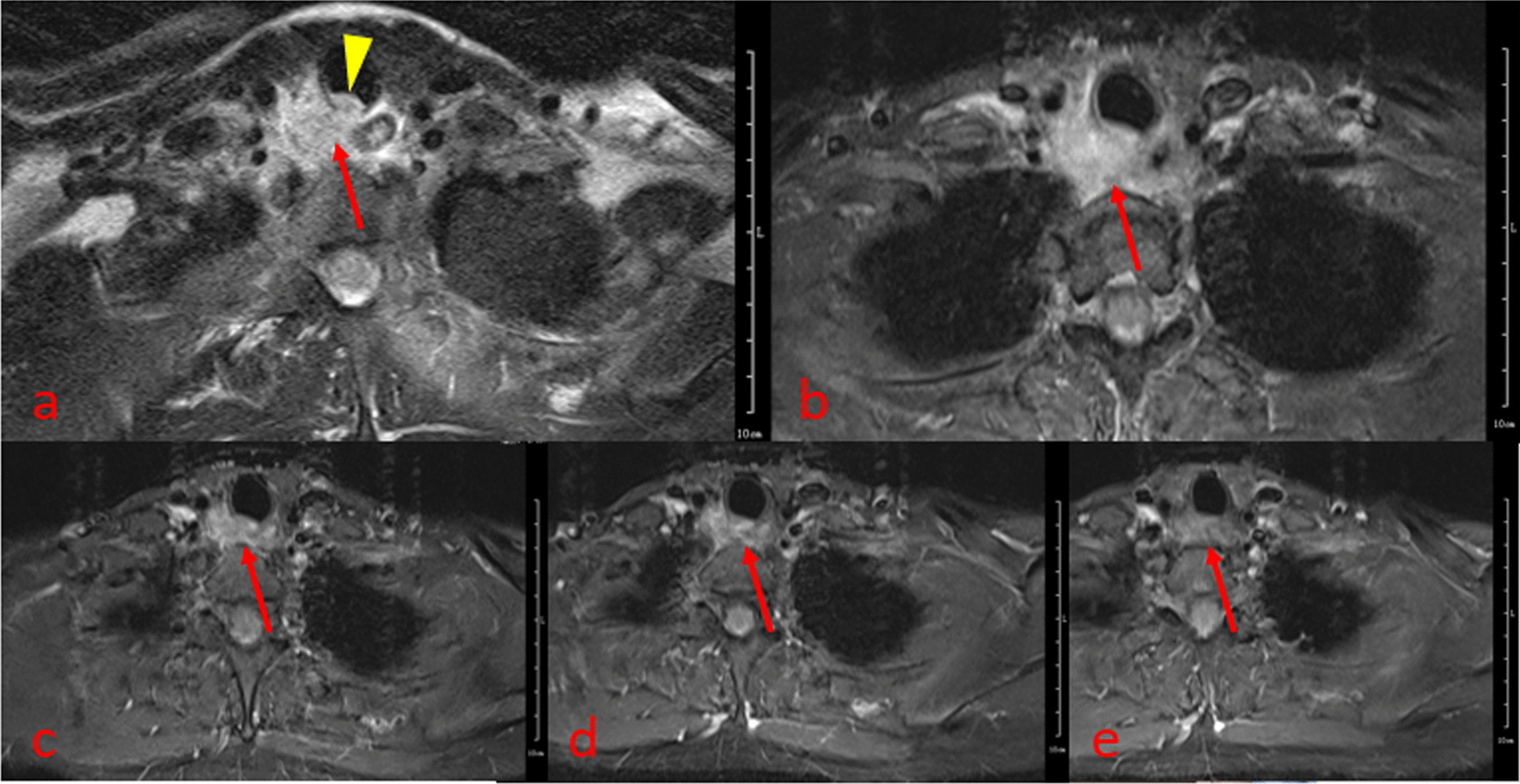
Axial T2-TIRM (**a**) showed a high signal mass behind the upper trachea (arrow) with an irregular and ill-defined margin. The sign that local thickening and signal increase of posterolateral wall of the upper trachea (arrowhead) suggests that the lesion may arise from the tracheal diverticulum. The esophageal wall was intact. The lesion gradually resolved, and the signal was gradually decreased after 2 days (**b**), 4 days (**c**), 7 days (**d**) and 21 days (**e**) of conservative treatment

The infected case performed a CT scan 1 year previously because of repeated bronchitis, by which a TD was detected (Fig. 3). Currently, the patient presented a paratracheal abscess. CT revealed a complex air- and fluid-filled mass in the right posterolateral tracheal wall, which had no enhancement after contrast injection (Fig. 4). CT identified no relationship between the abscess and the trachea. Meanwhile, MR imaging clearly showed an abnormal signal mass behind the upper trachea with an irregular and ill-defined margin. The posterolateral wall of the upper trachea was thickened, and the wall signal was high (Fig. 5a), which suggested that the abscess was associated with the trachea.

The patient symptoms gradually relieved, and the lesion showed gradual absorption after conservative treatment. MRI (T2-TIRM sequence) sensitively showed the absorption process of the lesion (Fig. 5b–e).

## Discussion

At present, multidetector CT scanning has been shown to be the most effective method for evaluating the presence and features of TD [14]. Characteristic imaging findings include a thin-walled air sac in the paratracheal area with or without communication to the tracheal lumen [1, 7]. The current study showed that MRI had lower capacity in detected the disorder, but could be used as a supplementary method for TD diagnosis. The general MRI finding is an oval or circular air-filled cystic lesion (no signal area) just behind the right side of the trachea in all sequences.

MRI can provide information about the wall of TD which cannot be detected by CT, because it has higher soft tissue resolution. Lesions measured by MRI appear larger than detected by CT, because the diameter measured by MRI is the outer diameter that includes the thickness of the wall, while CT measures the inner diameter. Nevertheless, MRI has reduced ability compared with CT in showing the communication between the air cyst and the trachea. One reason is that the connecting stalk often has a small size, while MR images have thick-slice and wide-intervals; in addition, some cases do not have a visible connecting stalk. Previous studies have shown that the connection between the air cyst and the trachea have different detectable rates between thick-section CT (> 1 mm) and thin-section CT (≤ 1 mm) images, and are detected more frequently with thin-section CT [1, 2, 8–10]. In the future, thin-section CT and three-dimensional MR sequences should be used to display the above connection and to compare the detection rate of thin-section CT.

Identifying the origin of a solitary neck abscess by CT is different, like in our case. On the one hand, the lesion was close to the posterior wall of trachea. On the other hand, the esophagus was partially surrounded by the lesion. In this study, MRI clearly showed a relationship between the abscess and the trachea. These findings indicate MRI is superior to CT in diagnosing infected TD presenting as a paratracheal abscess. The sign of local thickening and signal increase of the tracheal wall observed by MRI (T2-TIRM sequence) is the key for determining the origin of the lesion. This sign means that infection has spread into the trachea through the thin channel. To our knowledge, infected TD presenting as a paratracheal mass has only been reported in two patients [15, 16]. It is assumed that repeated upper airway inflammation produces mucus in the diverticulum, which then progresses to an abscess [15].

MRI plays an important role in monitoring therapeutic efficacy in infected TD, and should be the first choice in managing such lesions. Most TD cases are asymptomatic and discovered incidentally, not requiring clinical intervention [9, 17, 18]. Both CT and MRI can be used to follow-up these patients. However, in symptomatic patients requiring conservative medical management (antibiotics, mucolytic agents and physiotherapy) and close observation, MRI may be better than CT. Because MRI involves no radiation and has higher soft tissue resolution, it is more suitable for short-term multiple examinations. MRI can sensitively reveal the changes of the lesion, which may help clinicians judge whether the treatment plan is effective, enabling timely adjustment of the medication or treatment plan.

### Limitations

There were shortcomings in this study. We only analyzed the role of common MR sequences in diagnosing and managing TD, and special sequences, e.g., like thin-section and three-dimensional sequences, were not include in the study. We did not discuss the relationship between TD and lung diseases (e.g., chronic obstructive pulmonary disease). In addition, the study cohort was small, and a further study is needed.

### Conclusion

MRI can be used as a supplementary method for the diagnosis of TD, providing information about the lesion wall that cannot be detected by CT. MRI is superior to CT in diagnosing infected TD presenting as a paratracheal abscess, as well as monitoring therapeutic efficacy in infected TD.

## Data Availability

The datasets used and/or analysed during the current study are available from the corresponding author on reasonable request.

## Abbreviations

TD: Tracheal diverticulum
MR: Magnetic resonance
MRI: Magnetic resonance imaging
CT: Computed tomography
T2-TIRM: T2WI with turbo inversion recovery magnitude

## References

[1] Buterbaugh JE, Erly WK (2008). Paratracheal air cysts: a common finding on routine CT examinations of the cervical spine and neck that may mimic pneumomediastinum in patients with traumatic injuries. Am J Neuroradiol.

[2] Goo JM, Im JG, Ahn JM (1999). Right paratracheal air cysts in the thoracic inlet:clinical and radiologic significance. Am J Roentgenol.

[3] Ampollini L, Bobbio A, Carbognani P (2007). Incidental radiological finding of tracheal diverticulum. Eur J Cardiothorac Surg.

[4] Haghi Z, Towhidi M, Fattahi H (2009). Right paratracheal air cyst (tracheal diverticulum). Respir Care.

[5] Han S, Dikmen E, Aydin S (2008). Tracheal diverticulum: a rare cause of dysphagia. Eur J Cardiothorac Surg.

[6] Shah AR, Lazar EL, Atlas AB (2009). Tracheal diverticula after tracheoesophageal fistula repair: case series and review of the literature. J Pediatr Surg.

[7] Lin H, Cao Z, Ye Q (2014). Tracheal diverticulum: a case report and literature review. Am J Otolaryngol.

[8] Kim JS, Kim AY, Yoon Y (2011). Paratracheal air cysts using low-dose screening chest computed tomography: clinical significance and imaging findings. Jpn J Radiol.

[9] Cheng HM, Chang PY, Chiang KH, Huang HW, Lee CC (2012). Prevalence and characteristics of paratracheal air cysts and their association with emphysema in a general population. Eur J Radiol.

[10] Bae HJ, Kang EY, Yong HS, Kim YK, Woo OH, Oh YW, Doo KW (2013). Paratracheal air cysts on thoracic multidetector CT: incidence, morphological characteristics and relevance to pulmonary emphysema. Br J Radiol.

[11] Pace M, Dapoto A, Surace A, Di Giacomo A, Morzenti C, Costantini E, Sala F, Sironi S (2018). Tracheal diverticula: a retrospective analysis of patients referred for thoracic CT. Medicine (Baltimore).

[12] Balci NC, Noone T, Akün E, Akinci A, Klör HU (2003). Juxtapapillary diverticulum: findings on MRI. J Magn Reson Imaging.

[13] Morita S, Ueno E, Masukawa A, Suzuki K, Machida H, Fujimura M (2009). Defining juxtapapillary diverticulum with 3D segmented trueFISP MRCP: comparison with conventional MRCP sequences with an oral negative contrast agent. Jpn J Radiol.

[14] Soto-Hurtado EJ, Peñuela-Ruíz L, Rivera-Sánchez I (2006). Tracheal diverticulum: a review of the literature. Lung.

[15] Akabane S, Kawachi J, Fukai R, Shimoyama R, Kashiwagi H, Ogino H, Watanabe K (2016). A rare case of an infected tracheal diverticulum requiring emergency intervention: a case report. Int J Surg Case Rep.

[16] Charest M, Sirois C, Cartier Y, Rousseau J (2012). Infected tracheal diverticulum mimicking an aggressive mediastinal lesion on FDG PET/CT: an interesting case with review of the literature. Br J Radiol.

[17] Gayer G (2016). Tracheal diverticula. Semin Ultrasound CT MR.

[18] Inam H, Zahid I, Fatimi S (2019). Tracheal diverticulum as a rare cause of dysphagia. Asian Cardiovasc Thorac Ann.

